# The Problem of Advanced Consumption

**Published:** 1918-05-04

**Authors:** 


### The Hospital

The Workers' Newspaper of Administrative Medicine and Institutional Life, Administration, National Insurance and Health.

No. 1665, Vol. LXIV. SATURDAY, MAY 4, 1918. PRICE ONE PENNY

### THE PROBLEM OF ADVANCED CONSUMPTION.

This is a time to be practical and (at last, it is to be feared, for many people) candid. So we might as well begin, not at the beginning, but with present facts, and see what is actually done in the urgent matter of dealing with hopeless cases of pulmonary phthisis; which cases for present purposes may be divided into military and civil. Probably nearly every tuberculosis authority gives every preference it can to discharged soldiers with consumption. Its officers allow hardly any degree of disease, however severe, to deprive them of their chance of amelioration at the sanatorium, and keep civilian patients waiting until they are admitted. Some do not, however, choose to leave the freedom of home, and a few are too ill to travel any great distance, if at all: one or two of these the district council medical officer of health, if still out of the Army, may get into his isolation hospital for a time; a stray one gets into the workhouse and the papers. But ultimately a huge proportion of these poor fellows settles down to be ill at home, for better or worse. Owing to reasons already mentioned in these pages, they are very apt to leave sanatoriums of their own accord, or to be a little too much for the management of the institution, so that their departure therefrom is likewise premature. After absence from home they, like the rest of us, prefer to be with friends and acquaintances and in the familiar surroundings and circle of interests. Most of all is this the case if they feel themselves losing ground: nearly every man would rather die at home than away.

This last motive—so strong as it is—obviously operates also in the case of the civilian. But with him, unfortunately, there is only too often hardly any standing at all just now for those who would try to counteract it in the interests of the public health. "Hospital" as distinguished from "sanatorium" beds were never very numerous. The example set long ago by the Westmorland tuberculosis authorities at their institution at Meathop has been very inadequately followed. Like many pioneers, they have not "advertised." However, that is a digression; certainly it is not cause and effect. Now these scanty "hospital" beds, for advanced cases, with the extra nursing implied—terminal phthisis takes a vast deal of nursing—are at present practically all earmarked for soldiers, or else for cases like that of a phthisical mother of seven or eight young children, whose protection from infection is nothing less than imperative. And so the working man dying of consumption does it at home. Of course he should not, and were there more accommodation for him he would not so often. It is well to remember, however, that whatever alternative you could offer him he would still do it fairly often.

They say the medical profession is taking the place of the clerical one ("they" includes high scientific authorities like Rignano), so that no apology is needed for concluding with a moral. Besides, there is the word "problem" in the title, and in face of a problem something must be done. It is easy to raise the cry "Build hospitals!" How can any building, to count, be done now or for an uncertain time to come? And are those who raise the cry prepared to assent to the consequent swelling of their own proper income tax, which seems likely to surpass 50 per cent. already? How can more nurses be got—or doctors—or even, perhaps, drugs and rations? Let us not, then, disgust the practical politician, but advise increased accommodation when that becomes possible. The advent of a Ministry of Health might hasten this desirable time. As for filling the buildings when erected, and working them to the best advantage, it has to be said that with this aspect we encounter the pitiable drama of a dying man's last wishes threatening his child's life. If "nobody is to be forced to do anything," more particularly a dying man, then the argument cannot be completed, because if the consumptive has his way, then often enough his infant is going to be forced to die of tuberculous meningitis; always forced to run a big risk of so dying, or of getting a crop of tubercles in its little belly, or of other physical misfortune. We will not, however, imitate our predecessors in the pulpit and be over-dogmatic. Let public opinion—not that we respect it very greatly—choose. Marriages are free in England, arranged in France: married happiness is about the same in the two countries. But if medical authority were more real, men and women would be healthier, and thereby—necessarily, is it not?—so much the happier and better off. After the war someone must write an anti-tuberculosis propaganda play.

